# Epstein-Barr virus encoded microRNA BART7 regulates radiation sensitivity of nasopharyngeal carcinoma

**DOI:** 10.18632/oncotarget.15526

**Published:** 2017-02-20

**Authors:** Wei Gao, Zeng-Hong Li, Siqi Chen, Jimmy Yu-Wai Chan, Min Yin, Min-Juan Zhang, Thian-Sze Wong

**Affiliations:** ^1^ Department of Surgery, The University of Hong Kong, Hong Kong SAR; ^2^ Shenzhen Institute of Research and Innovation, The University of Hong Kong, Guangdong Province, China; ^3^ Department of Otolaryngology, The First People's Hospital of Foshan, Guangdong Province, China; ^4^ Department of Otorhinolaryngology Surgery, The First Affiliated Hospital of Nanjing Medical University, Nanjing, China

**Keywords:** nasopharyngeal carcinoma, EBV, radiation sensitivity, MicroRNA BART7

## Abstract

Epstein-Barr virus (EBV)-associated nasopharyngeal carcinoma (NPC) is very sensitive to radiotherapy. To date, the underlying mechanism remains poorly understood. Here, we demonstrated that expression of EBV-encoded microRNA BART7 (ebv-miR-BART7) increases responsiveness of NPC to radiation treatment by targeting GFPT1/TGFβ1 signaling. GFPT1 is the the key rate-limiting enzyme of the hexosamine signaling pathway and governs TGFβ1 production. TGFβ1, a pleotropic cytokine with the potency to trigger self-renewal and damage-repair machinery in somatic cells. TGFβ1 can protect zebrafish embryo from the lethal effects of radiation treatment. In silico analysis showed that ebv-miR-BART7 could target GFPT1 transcript. Correlation analysis on primary NPC tissues suggested that ebv-miR-BART7 and GFPT1 have negative expression correlation. Expression of GFPT1 and TGFβ1 were inducible by radiation in NPC cell with ebv-miR-BART7 expression. Further, suppressing endogenous GFPT1 expression inhibited TGFβ1 which subsequently increased the responsiveness of NPC to radiation treatment. Taken together, our results demonstrated that ebv-miR-BART7 controls TGFβ1 production by targeting GFPT1. Detection of ebv-miR-BART7 may provide useful indicator for monitoring NPC progression and predict therapeutic outcomes.

## INTRODUCTION

Nasopharyngeal carcinoma (NPC) is endemic in Southeast Asia and southern China [1, 2]. Guangdong province in the southern China has the highest incidence worldwide [3]. Histologically, NPC are classified into 3 sub-types including keratinizing squamous-cell carcinoma (type I), non-keratinizing squamous carcinoma (type II) and undifferentiated carcinoma (type III) [2]. Treatment for NPC is primarily based on radiotherapy [2]. Efficacy of radiotherapy or sensitivity of NPC to radiotherapy will have a direct impact on treatment outcome [4, 5]. Failure of radiotherapy will lead to disease recurrence or metastasis [6]. Despite of the encouraging results of NPC radiotherapy, recurrence of NPC after initial treatment remains obvious in particular regions with incidence ranging from from 8%-58% [5]. So far, the mechanisms governing the sensitivity of NPC to radiotherapy remains poorly understood.

NPC is characterized by the close association with Epstein-Barr virus (EBV) infection and the oncogenic functions of EBV is well documented [7]. Recently, it was found that the small microRNA encoded by the EBV genome is a new class of oncogenic promoter which plays a key role in tumor development. MicroRNA did no code for protein. Mature microRNA inhibits protein expression by promoting target mRNA degradation and/or inhibit the translation process [8]. Deep sequencing analysis identified 44 EBV-encoded microRNA in the NPC tissues [9]. Our previous study revealed that ebv-miR-BART7 was expressed at high level in the undifferentiated NPC tissues. In addition, cell-free circulating ebv-miR-BART7 was detectable in the peripheral blood of NPC patients [10, 11]. Patients with ebv-miR-BART7 positive margins had a significantly higher chance of developing local tumor recurrence [12]. The data reveals that ebv-miR-BART7 is important in the pathogenesis of NPC.

NPC cells expressing ebv-miR-BART7 is more susceptible to radiation treatment [11]. Here, we demonstrated that expression of EBV-encoded microRNA BART7 (ebv-miR-BART7) increases responsiveness of NPC to radiation treatment by targeting GFPT1/TGFβ1 signaling. GFPT1 is the the key rate-limiting enzyme of the hexosamine signaling pathway and governs TGFβ1 production [13]. TGFβ1, a pleotropic cytokine with the potency to trigger self-renewal and damage-repair machinery in somatic cells [14]. In silico analysis showed that ebv-miR-BART7 could target GFPT1 transcript. Correlation analysis on primary NPC tissues suggested that ebv-miR-BART7 and GFPT1 have negative expression correlation. Expression of GFPT1 and TGFβ1 were inducible by radiation in NPC. The data indicated that ebv-miR-BART7 could possibly mediate TGFβ1 production by targeting GFPT1.

## RESULTS

### GFPT1 is a target gene of ebv-miR-BART7

We hypothesized that ebv-miR-BART7 expressing NPC response differently as compared with the ebv-miR-BART7 negative NPC after radiation treatment. Thus, we employed microarray to evaluate the transcriptomic changes of ebv-miR-BART7 expressing HONE1 and the mock transfectant after receiving cellular irradiation at 4 Gy. As microRNA is a negative gene regulator, we only focus on genes which showed differential reduced after irradiation. Using fold change 1.5-fold and *P <* 0.05 as cut-off, 73 genes were significantly reduced in the ebv-miR-BART7 expressing HONE1 after receiving radiation treatment.

**Figure 1:**
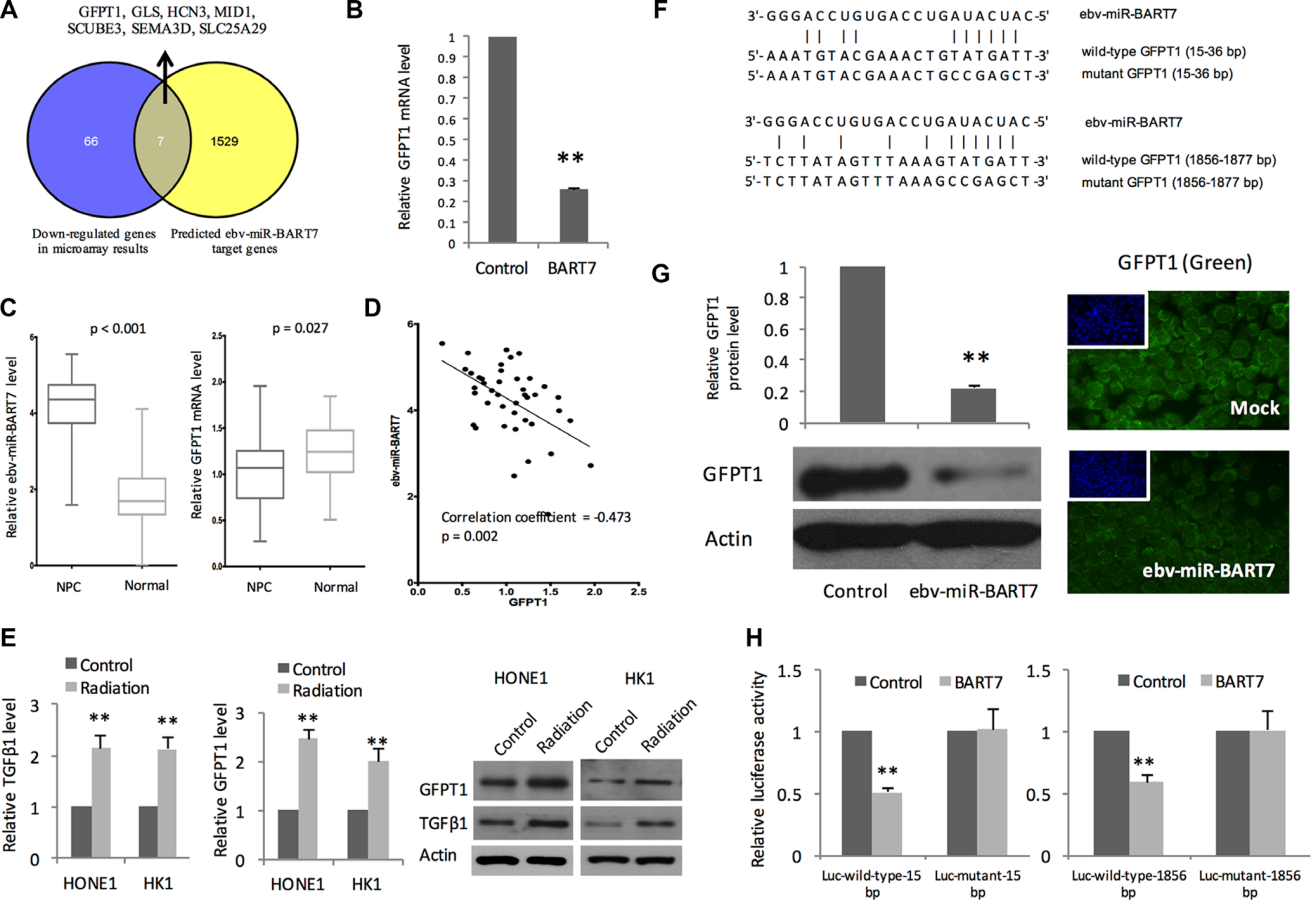
EBV-encoded microRNA BART7 targeting GFPT1 in NPC (**A**) Integration of microarray results and computational prediction identified ebv-miR-BART7 target genes. GFPT1 was significantly reduced in ebv-miR-BART7 expressing HONE1 after irradiation at 4 Gy. Computation prediction using ebv-miR-BART7 seed sequence suggested that GFPT1 transcript could form thermodynamically stable duplex with ebv-miR-BART7; (**B**) Reduced GFPT1 in irradiated HONE1 is confirmed by QPCR analysis. GAPDH was used as internal controls; (**C**) Expression level of ebv-miR-BART7 and GFPT1 between primary NPC (*n* = 42) and healthy controls (*n* = 29) were shown in the box plots. Elevated ebv-miR-BART7 was found in the primary NPC tissues without subjected to radiotherapy treatment. In comparison with the normal nasopharyngeal epithelia, GFPT1 level was significantly reduced in NPC; (**D**) Significant negative correlation was observed between ebv-miR-BART7 and GFPT1 level in NPC tissues; (**E**) QPCR and Western blot analysis showed that GFPT1 and TGFβ1 level was elevated in NPC cells following radiation at 4 Gy; (**F**) Predicted ebv-miR-BART7 binding sites on the 3′-UTR of GFPT1 mRNA transcript; (**G**) Western blot and immunostaining analysis showed that GFPT1 protein was significantly reduced in HONE1 cells transfected with ebv-miR-BART7 mimic; (**H**) Results of luciferase reporter assay showed that ebv-miR-BART7 binds to the 2 sites on GFPT1 transcript. Two predicted regions of wild-type and mutant 3′-UTR of GFPT1 were cloned into pMIR-REPORT Luciferase vector to generate Luc-wild-type vector and Luc-mutant vector, respectively. Then. HONE1 cells were co-transfected with Luc-wild-type vector or Luc-mutant vector, BART7 mimic or negative control and pMIR-REPORT β-galactosidase control vector. Tansfected cells were measured for changes in firefly luciferase and β-galactosidase activities. **P < 0.01.

On the other hand, we performed computational prediction using Vir-Mir db to identify potential ebv-miR-BART7 target genes. Minimum free energy (mfe) was used to evaluate the stability of ebv-miR-BART7/mRNA duplex. Using -25.0 kcal/mol mfe as cut-off, 1536 genes were identified as potential target genes of ebv-miR-BART7. Of which, 7 genes (GFPT1, GLS, HCN3, MID1, SCUBE3, SEMA3D, SLC25A29) matched with the gene list obtained from the microarray experiments (Figure 1A). QPCR analysis showed that ebv-miR-BART7 expressing HONE1 had a significant reduction of GFPT1 transcript level (Figure 1B). To corroborate the findings, we measured ebv-miR-BART7 and GFPT1 transcript level in primary NPC tissues (prior treatment) and normal nasopharyngeal epithelia (Figure 1C). In the NPC tissues, ebv-miR-BART7 was expressed at high levels (*P <* 0.001). In contrast, GFPT1 was significantly reduced in comparison with the normal counterparts (*p* = 0.027). Furthermore, as shown in Figure 1D, expression of ebv-miR-BART7 and GFPT1 showed negative correlation (Correlation coefficient = -0.47, *p* = 0.002). In ebv-miR-BART7 negative NPC cell lines, both mRNA and protein expression levels of GFPT1 and the downstream regulated gene TGFβ1 were induced in response to radiation treatment at the dose of 4 Gy (Figure 1E).

Sequence analysis indicated that the seed sequence of ebv-miR-BART7 could bind to 3′UTR of GFPT1 at 2 sites: 15-36 and 1856-1877 (Figure 1F). In HONE1, restored ebv-miR-BART7 in the EBV-negative cell line using synthetic ebv-miR-BART7 mimic could reduce GFPT1 protein level (Figure 1G). To further confirm the post-transcriptional regulatory role of ebv-miR-BART7 on GFPT1, we constructed luciferase reporter constructs containing either wild-type or mutant 3′untranslated region (UTR) of GFPT1 and transfected into HONE1 cells. If ebv-miR-BART7 could target the predicted sites, transfection of ebv-miR-BART7 mimics shall bind and reduce the luciferase activity. As shown in Figure 1H, transfection of ebv-miR-BART7 mimic decreased the luciferase activity in cells with wild-type transcript. As the ebv-miR-BART7 are not specific to the binding sites of mutant construct, the inhibitory effect on luciferase activity was not observed in cell transfected with mutant luciferase reporter constructs.

### GFPT1 knockdown reduces TGFβ1 production by NPC

**Figure 2:**
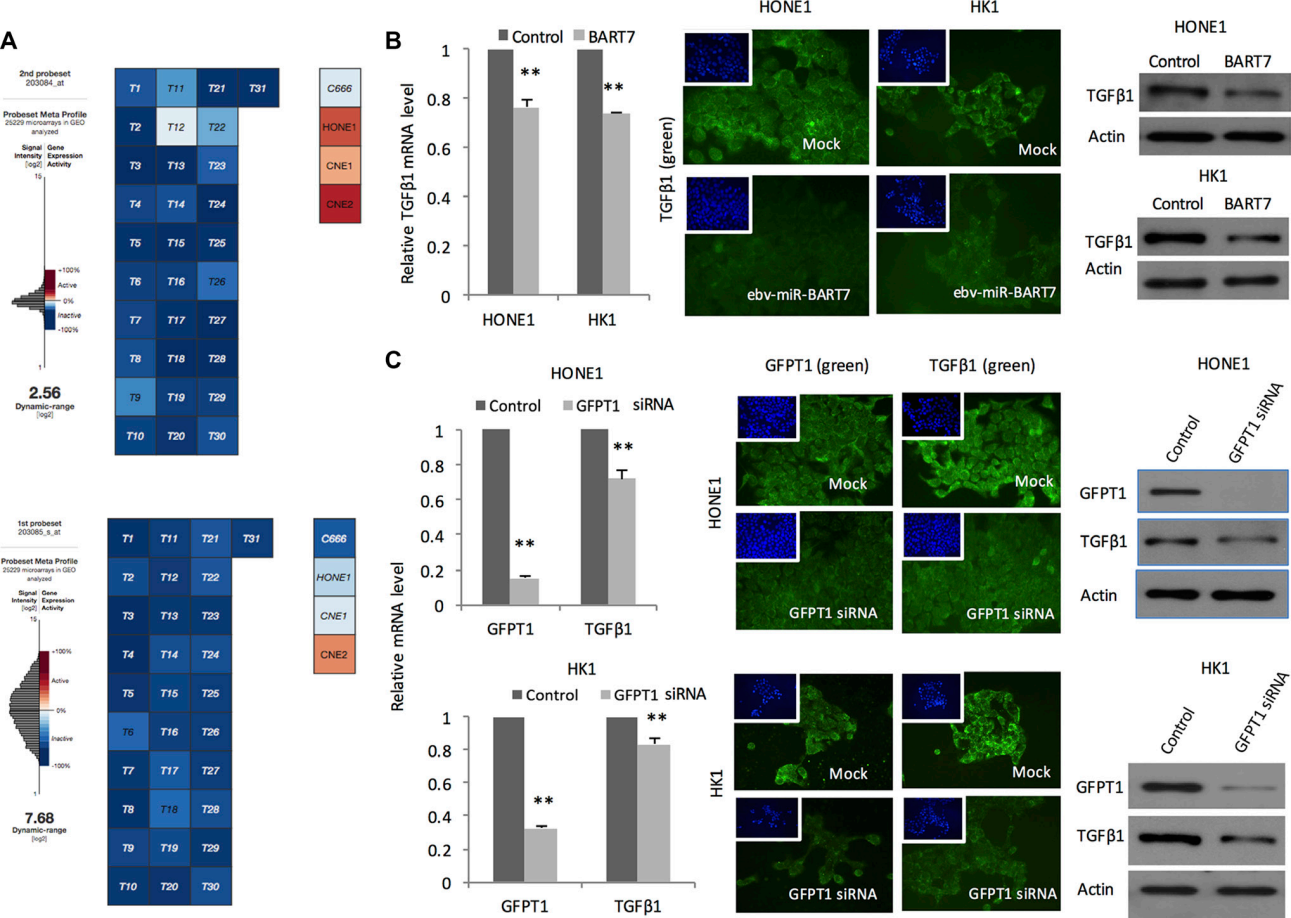
Expression of TGFβ1 and the association with ebv-miR-BART7 and GFPT1 (**A**) Absolute expression level of TGFβ1 revealed by microarray datasets obtained from GEO; (**B**) QPCR, Western blot and immunostaining of TGFβ1 level in NPC cells transfected with ebv-miR-BART7 mimics showed significant reduction in both RNA and protein levels; (**C**) Expression reduction of TGFβ1 in NPC cells transfected with GFPT1 siRNA. **P < 0.01.

EBV is present in all the undifferentiated NPC tissue samples. In comparison, EBV genome and the viral gene products is lost during continued passaging in most of the cell line models. At present, C666 is the only established NPC cell line harboring the viral genome and expressing ebv-miR-BART7 [15]. To explore the role of EBV in mediating TGFβ1 production, we extracted the absolute expression value of TGFβ1 in NPC tissues and cell lines in microarray datasets in public domains. All the EBV containing samples including primary NPC tissues and C666 demonstrated low expression level of TGFβ1. In contrast, the EBV-negative cells (HONE1, CNE1, & CNE2) had remarkable higher TGFβ1 expression (Figure 2A). To examine whether the reduced TGFβ1 in EBV-positive samples was attributed to ebv-miR-BART7, we examined the expression changes of TGFβ1 in NPC cells after transfection with ebv-miR-BART7 mimics. Significant reduction in TGFβ1 mRNA and protein level were observed in the transfectants (Figure 2B). In addition, TGFβ1 level in EBV-negative cells could also be suppressed with the use of GFPT1 siRNA (Figure 2C). Overall, the data suggested that ebv-miR-BART7 inhibited TGFβ1 production by targeting GFPT1.

### TGFβ1 confers radiation protection to NPC

**Figure 3:**
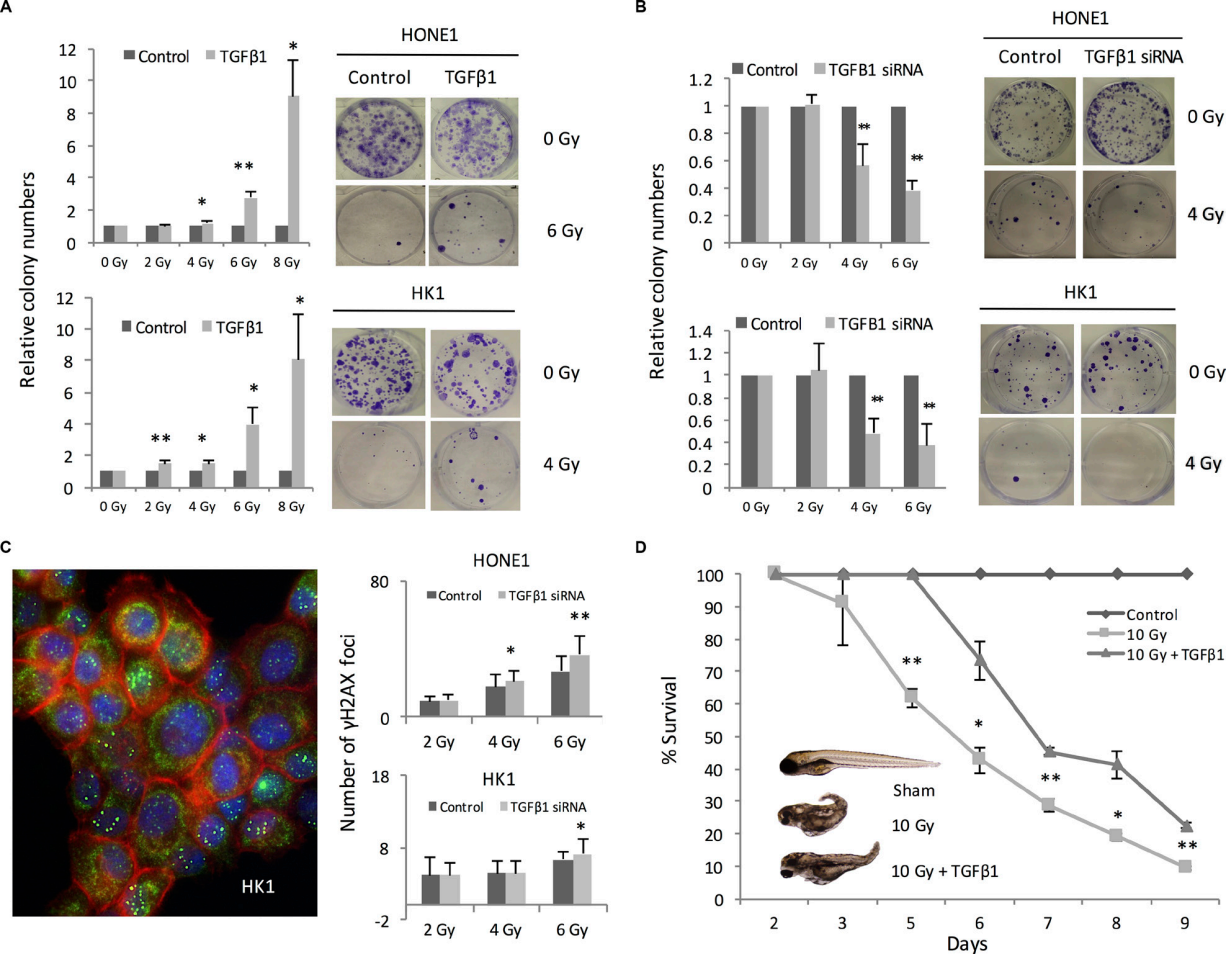
TGFβ1 conferred radiation protection to NPC cells (**A**) Colony formation in NPC cells pre-treated with recombinant TGFβ1 before irradiation; (**B**) Reduced colony formation ability in TGFβ1 siRNA transfectant following radiation treatment; (**C**) TGFβ1 knockdown using siRNA was performed in HONE1 and HK1 cells. The number of rH2AX foci formation of HONE1 and HK1 cells in the irradiated cells were counted; (**D**) TGFβ1 pre-treatment improved survival of zebarfish embryo receiving radiation treatment. *P < 0.05; **P < 0.01.

Having demonstrated the relationship between ebv-miR-BART7 and TGFβ1, we tested whether TGFβ1 could affect the sensitivity of NPC cells to radiation. NPC cells were treated with recombinant TGFβ1 before radiation treatment. HONE1 was treated with recombinant TGFβ1 at 10 ng/ml. For HK1, the cells were incubated with TGFβ1 at 0.5 ng/ml because TGFβ1 higher than this level could had growth inhibitory effect (data not shown). The colony forming ability of pre-treated cells was higher in comparison with the parallel control. In addition, the colony forming ability is remarkable higher in the pre-treated cells exposing to high-dose radiation (Figure 3A). To confirm the results, radiation treatment was repeated on NPC cells transfected with TGFβ1 siRNA. Significantly reduction in the number of colony following radiation was observed in both HONE1 and HK1 cells transfected with TGFβ1 siRNA (Figure 3B). In addition, TGFβ1 knockdown cells were more susceptible to radiation-induced DNA damages. In comparison with the cells transfected with scramble siRNA, the number of γH2AX foci increased in the TGFβ1 knockdown cells in a dose-dependent manner (Figure 3C). To confirm the protective effect of TGFβ1 *in vivo*, we examined the lethal effects of radiation on zebrafish embryos with or without pre-incubation with TGFβ1. The percentage survival of the zebrafish embryos was significantly reduced in comparison with the sham control. In the group with TGFβ1 pre-treatment, radiation-induced death was also observed. However, the percentage survival of the embryos was significantly higher as compared with the group without receiving TGFβ1 pre-treatment (Figure 3D).

### TGFβ1 impacts on proliferation, apoptosis, and autophagy following irradiation

**Figure 4:**
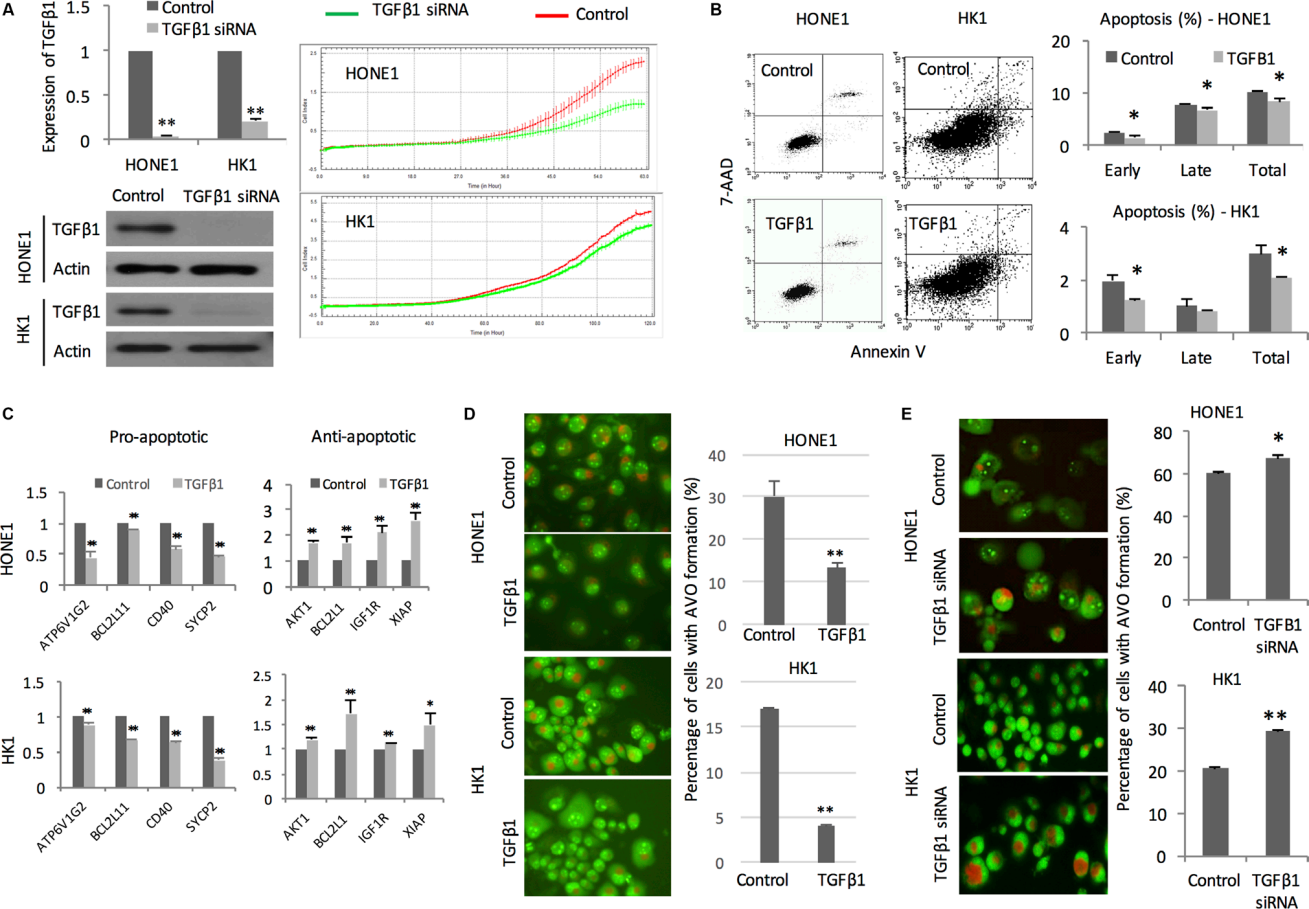
TGFβ1 impacts on proliferation, apoptosis, and autophagy (**A**) QPCR and Western blot analysis showing TGFβ1 mRNA levels in HONE1 and HK1 cells transfected with TGFβ1 siRNA. Proliferation kinetics of NPC cells transfected with TGFβ1 siRNA and mock control after receiving radiation treatment was measured continuously by xCELLigence Real-Time Cell Analyzer; (**B**) Representative dot plots of apoptotic cells of irradiated HONE and HK1 with or without recombinant TGFβ1 pre-treatment were analyzed by flow cytometry; (**C**) PCR array showing relative mRNA expression level of pro-apoptotic and anti-apoptotic genes in irradiated HONE1 and HK1 cells. GAPDH was employed as internal control; (**D**) AVO formation in NPC cells pretreated with recombinant TGFβ1 under exposure to radiation. Percentage of cells with AVO formation was calculated; (**E**) AVO formation in NPC cells transfected with TGFβ1 siRNA upon radiation treatment. *P < 0.05; **P < 0.01.

Real-time cellular kinetic study showed that TGFβ1 knockdown cells had reduced proliferation capacity in comparison with the mock transfectant following radiation (Figure 4A). Recombinant TGFβ1 treatment decreased the percentage of apoptotic cells induced by radiation (Figure 4B). We attempted to identify apoptosis-related genes using Cell-Death PCR Array. We followed the gene selected by the Cell Death PathwayFinder (https://www.qiagen.com/hk/shop/pcr/primer-sets/rt2-profiler-pcr-arrays?catno=PAHS-212Z#geneglobe) and test the gene expression changes in the NPC cells. In the presence of TGFβ1, the pro-apoptotic genes (ATP6VIG2, BCL2L11, CD40, SYCP2) were significantly reduced after irradiation. On the other hand, the anti-apoptotic genes (AKT1, BCL2L1, IGFIR, XIAP) were increased significantly after irradiation (Figure 4C). Formation of acidic vesicular organelles (AVO) after radiation is another indicator of radiation sensitivity of cancer cells [16]. Cells pretreated by TGFβ1 exhibited significantly lower number of AVO implicating that they are more resistant to radiation (Figure 4D). In transfectant containing TGFβ1 siRNA, significantly higher number of AVO suggested that they were more sensitive to radiation treatment (Figure 4E).

### GFPT1 knockdown sensitizes NPC cells to radiation treatment

**Figure 5:**
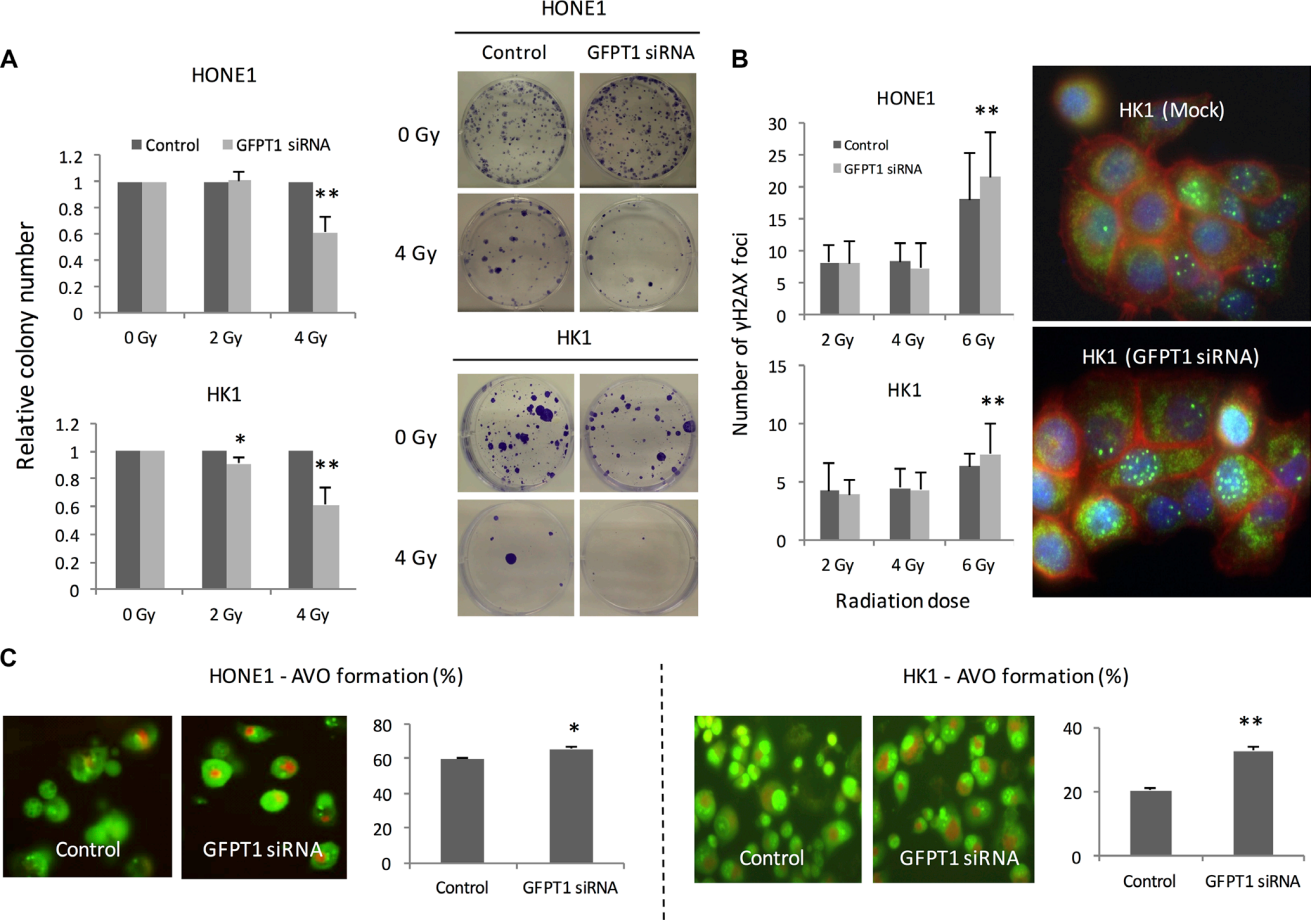
Knockdown GFPT1 sensitized HONE1 and HK1 cells to radiation (**A**) Colony formation ability of the irradiated HONE1 and HK1 cells transfected with GFPT1 siRNA; (**B**) rH2AX foci formation in nuclei of NPC cells transfected with GFPT1 siRNA; (**C**) Representative photos showed formation of acidic vesicular organelles (AVO) of HONE1 and HK1 cells transfected with GFPT1 siRNA upon radiation treatment. *P < 0.05; **P < 0.01.

As shown in Figure 1E, GFPT1 expression was induced following radiation treatment in NPC cells without ebv-miR-BART7 expression. Irradiated GFPT1 knockdown NPC cells resulted in low number of colony in both HONE1 and HK1 cells (Figure 5A). GFPT1 knockdown cells had a higher number of γH2AX foci as opposed to the mock control suggesting that more DNA double-strand break was induced by radiation (Figure 5B). In addition, silence of GFPT1 in NPC cells increased the percentage of cells that exhibited autophagy under radiation treatment (Figure 5C).

### Ebv-miR-BART7 regulates GFPT1-TGFβ1 pathway and enhances radiation sensitivity

**Figure 6:**
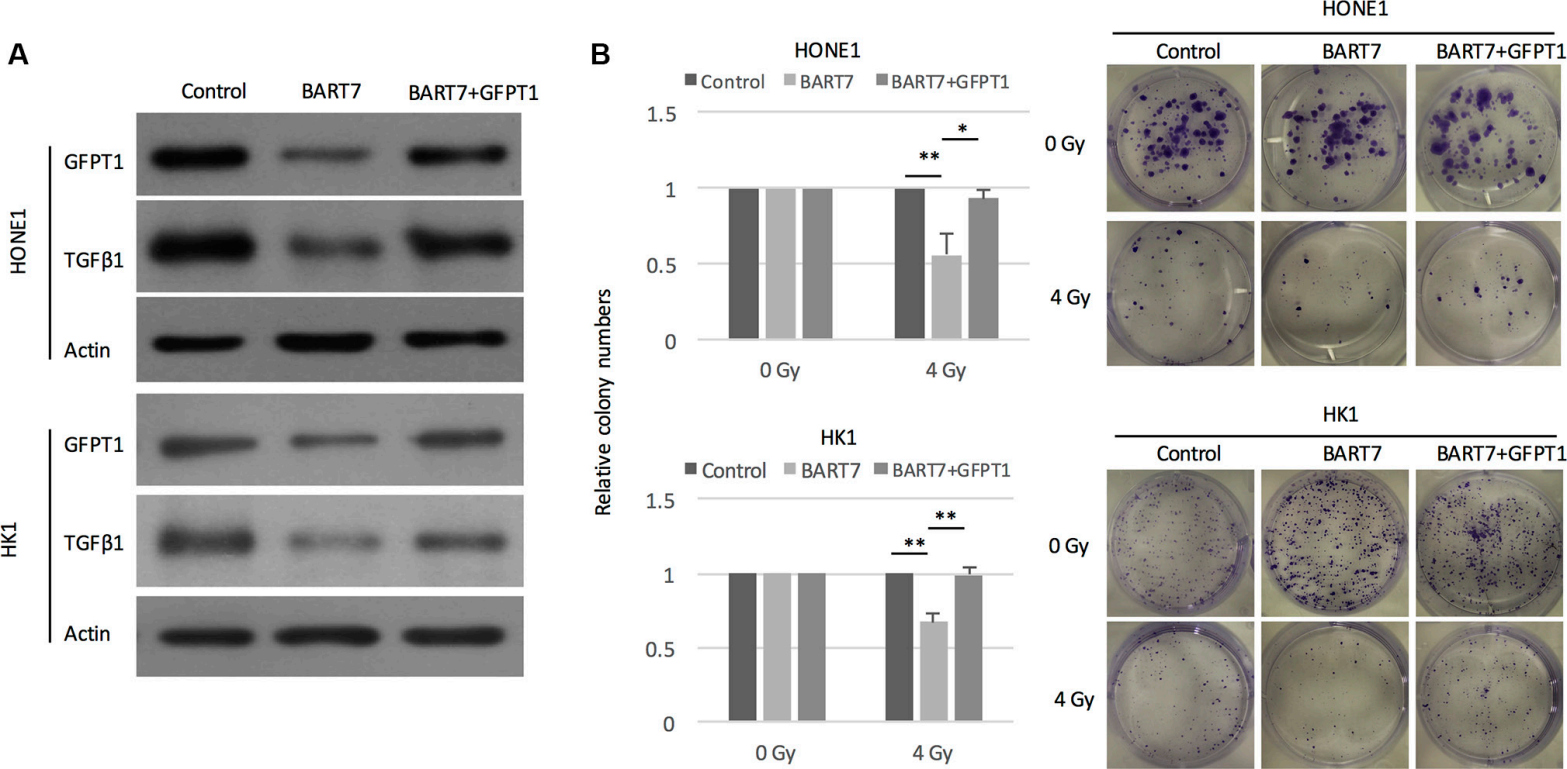
Ebv-miR-BART7 enhanced radiosensitivity by targeting GFPT1-TGFβ1 pathway (**A**) Western blot analysis showing protein expression level of GFPT1 and TGFβ1 in NPC cells transfected with ebv-miR-BART7 mimic with or without GFPT1 overexpression vector. (**B**) Colony formation of NPC cells transfected with ebv-miR-BART7 mimic with or without GFPT1 overexpression vector under exposure to radiation. *P < 0.05; **P < 0.01.

First, we transfected ebv-miR-BART7 into NPC cell lines and measured the changes of GFPT1 and and TGFβ1 protein. Western blot data shown that GFPT1 and TGFβ1 protein level was significantly reduced in ebv-miR-BART7 expressing HONE1 and HK1 cells (Figure 6A). The suppressing effects of ebv-miR-BART7 on GFPT1 and and TGFβ1 was reduced in NPC cells containing GFPT1 over-expressing vector (Figure 6A). Next, we attempted to evaluate whether the increased in radiation sensitivity contributed by ebv-miR-BART7 in NPC cells could be rescued in the GFPT1-overexpressing NPC cell lines. As shown in Figure 6B, the colony forming ability of NPC cells transfected with ebv-miR-BART7 mimics was significantly reduced after irradiation. The colony forming ability was increased in the GFPT1 overexpressing cells. Taken together, the data suggested that ebv-miR-BART7-GFPT1-TGFβ1 pathway is controlling radiation sensitivity in NPC cells.

## DISCUSSION

As EBV-positive NPC is more responsive to radiotherapy, we suggested that expression of EBV-encoded microRNA is functionally important to the sensitivity of NPC cells to radiation treatment. Ebv-Mir-BART7 is encoded by the BamHI A cluster in the EBV viral genome. High expression of ebv-miR-BART7 is noticed in the primary NPC tissues and circulation of NPC patients [17, 18]. Further, ebv-miR-BART7 expression is also found in other EBV-associated malignancies such as gastric carcinoma [19].

TGFβ1 is a pleotropic cytokine with the potency to trigger self-renewal and damage-repair machinery. Our data revealed that the proliferative inhibitory effects of radiation on NPC is prominently reduced in the presence of TGFβ1. Moreover, TGFβ1 knockdown NPC cells is more susceptible to radiation-induced DNA damages. TGFβ1 expression is associated with local tumor relapse and survival of NPC patients [20]. TGFβ1 could reduce ionizing radiation (IR)-induced DNA damage [21]. In mink lung epithelial cell line, activation of the TGFβ1 signaling cascade with exogenous TGFβ1 could inhibit apoptosis induced by gamma-irradiation [22]. Hence, tumour microenvironment with high TGFβ1 content shall favour NPC escape from the “killing effect” of exogenous radiation treatment.

TGFβ1 production is accompanied with increased activity in hexosamine biosynthesis pathway. The end product of hexosamine pathway, UDP-GlcNAc, modulates transcription regulators by O-glycosylation at specific activating sites (serine or threonine residues). Multiple glycosylated proteins are reported to be involved in TGFβ1 up-regulation. Glycosylated Sp1 could activate TGFβ1 expression by increasing the promoter activity. In addition, glycosylated PKCB could activate MAPK signalling cascade by stimulating p38 MAPK phosphorylation which will subsequently bind to TGFβ1 promoter and activate TGFβ1 transcription [23, 28]. In mesothelial cells, glucose functions as a strong effector in modulating TGFβ1 upregulation [24]. Therefore, controlling the rate-limiting enzyme of hexosamine pathway could regulate TGFβ1 expression in NPC.

Using PCR array, we demonstrated that TGFβ1 is involved in the radiation responses by suppressing the pro-apoptotic genes in the NPC. The association of these pro-apoptotic genes/ apoptotic-related genes with radiation responses has been demonstrated in several cancer models. For example, BCL2L11 (BIM) can regulate UV-induced apoptosis by controlling BAX activation [25]. Activated CD40 receptor can increase gamma-radiation induced apoptosis in multiple myeloma and B-lymphoma [26]. Inhibiting the AKT-regulated genes such as extra domain A (EDA) could enhance the radiation sensitivity of NPC cells [27]. High BCL2L1 expression is inversely correlated with the complete remission of oropharyngeal carcinoma patient treated with radical irradiation [28]. X-linked inhibitor of apoptosis protein (XIAP) can inhibit caspase-3, caspase-7, and caspase-9. In NPC cells, XIAP expression is induced by cell irradiation in a dose-dependent manner [29]. Thus, suppressing TGFβ1 expression could target multiple radiation responsive signaling cascade in NPC cells and mediate the sensitivity of cancer cells to radiation treatment.

Our findings established for the first time that ebv-miR-BART7 suppressed the expression of GFPT1 and TGFβ1 that could reduce the sensitivity of NPC cells to radiation treatment. Targeting GFPT1 could suppress TGFβ1 production and increase the radiation sensitivity of NPC cells. Targeting GFPT1 and TGFβ1 may be a new strategy to enhance the radiosensitivity of NPC and improve treatment efficacy. Further longitudinal study is warranted to establish the clinical association between ebv-miR-BART7 and the radiosensitivity or prognosis of NPC patients.

## MATERIALS AND METHODS

### Cell cultures

NPC cell lines HONE1 and HK1 were cultured in RPMI-1640 medium supplemented with 10% fetal bovine serum, 200 Unit/ml penicillin G sodium, 200 μg/ml streptomycin sulfate, and 0.5 μg/ml amphotericin B. HONE1 was established from a poorly differentiated nasopharyngeal carcinoma [30]. HK1 was derived from a well-differentiated nasopharyngeal carcinoma [31]. Cells were incubated at 37°C in a humidified incubator with 5% CO_2_. Cell irradiation was carried out by Gammacell^®^ 3000 Elansystem (Best Theratronics Ltd.).

### Clinical samples

Primary NPC and normal nasopharyngeal epithelia were collected from the Department of Surgery, The University of Hong Kong, Queen Mary Hospital, Hong Kong. Institutional Review Board of the hospital has approved this collection protocol (registered number: UW 10-142). For 42 patients with primary NPC, there were 32 males and 10 females. Their ages ranged from 12-75 years. For the 29 healthy volunteers, there were 20 males and 9 females and their ages ranged from 6–81 years.

### Microarray and in silico analysis

The RNA quality assessment and gene expression microarray were performed in the Genome Research Centre of the University of Hong Kong. GeneChip Human Genome U133 Plus 2.0 Array (Affymetrix) was employed to examine global gene expression profiling. Agilent 2100 bioanalyzer (Agilent Technologies) was used to evaluate RNA quality. GeneSpring GX version 10.0 (Agilent Technologies) was employed to analyze microarray data. The microarry data have been deposited in NCBI's Gene Expression Omnibus and are accessible through GEO Series accession number GSE79571. Microarray data of 31 NPC tissues were obtained from GSE12452 of GEO. Microarray data of HONE1, CNE1, CNE2 were obtained from GSE15047. Raw microarray data of C666-1 were obtained from GSE34573. All the microarray data were normalized and analyzed using Gene Expression Commons [32]. Targets of ebv-miR-BART7 was predicted using Vir-Mir db [33]. The minimum free energy (mfe) of the miRNA/mRNA duplex was calculated and a lower mfe indicated greater stability of the miRNA/mRNA duplex.

### RNA extraction and real-time quantitative RT-PCR (QPCR) analysis

Total RNA was extracted and purified using TRIZOL (Life Technologies). High Capacity cDNA Reverse Transcription Kit (Life Technologies) was used for cDNA synthesis. Primers and probes for QPCR were designed by Universal ProbeLibrary Assay Design Center (http://www.roche-applied-science.com/). Transcript levels were determined by qPCR on a LightCycler^®^ 480 (Roche Applied Science). Table 1 listed the sequence of primers and probes used in in the current study.

**Table 1:** Primer and probe sequences for QPCR

Gene	Forward primer (5′–3′)	Reverse primer (5′–3′)	Probe number*
ABL1	GGGCTGCAAATCCAAGAAG	ATGCTACTGGCCGCTGAA	78
AKT1	CCAGCCTGGGTCAAAGAAG	CTGGCCACAGCCTCTGAT	16
APAF1	CCTGTTGTCTCTTCTTCCAGTGT	AAAACAACTGGCCTCTGTGG	39
ATP6V1G2	AAGACCCAGGGGTAGTGGAG	CCCCCACCGCTTACTTCT	78
BAX	ATGTTTTCTGACGGCAACTTC	ATCAGTTCCGGCACCTTG	57
BCL2	AGTACCTGAACCGGCACCT	GCCGTACAGTTCCACAAAGG	75
BCL2A1	CAGGAGAATGGATAAGGCAAA	CCAGCCAGATTTAGGTTCAAA	75
BCL2L1	AGCCTTGGATCCAGGAGAA	AGCGGTTGAAGCGTTCCT	66
BCL2L11	GGCCCCTACCTCCCTACA	GGGGTTTGTGTTGATTTGTCA	21
BIRC2	GATATTGTGTCAGCACTTCTTAATGC	TCTGTTCTTCCGAATTAATGACAA	35
BIRC3	GCCCAGGAGATGAAAATGC	CATGATTGCATCTTCTGAATGG	80
CASP1	CCAGGACATTAAAATAAGGAAACTGTA	CCAAAAACCTTTACAGAAGGATCTC	1
CASP2	GGGGTCTTGGTCCACCTT	GCCACACACTCCCAATATCC	17
CASP3	TGTGAGGCGGTTGTAGAAGA	GGGCTCGCTAACTCCTCAC	76
CASP6	GATGCAGCCTCCGTTTACA	CACAGTTTCCCGGTGAGAATA	40
CASP7	CCGAGACTTTTAGTTTCGCTTT	CCTGATCATCTGCCATCGT	57
CASP9	GAACATCTTCAATGGGACCAG	CAAACCCATGGTCTTTCTGC	64
CD40	GGTCTCACCTCGCTATGGTT	CAGTGGGTGGTTCTGGATG	34
CD40LG	TCATGAAAACGATACAGAGATGC	CTTCGTCTCCTCTTTGTTTAACATT	2
CFLAR	CACCCAGATTGAGGATGGTC	GCTGCCAAAGGAGATTTAACA	48
CYLD	TTTGCGTGTGTTGAAAGTACAAT	TTCCTGCGTCACACTCTCTG	33
DFFA	CGAGCCACATCCTTACTGC	TTTGGGGTCTTCCTTGGTAA	7
FAS	GTGGACCCGCTCAGTACG	TCTAGCAACAGACGTAAGAACCA	60
FASLG	CAGTCCACCCCCTGAAAAA	GGACCTTGAGTTGGACTTGC	73
GADD45A	GGAGAGCAGAAGACCGAAAG	AGTGATCGTGCGCTGACTC	37
GFPT1	AGCTGTGCAAACACTCCAGA	TTCCTTCTGCATAAATGAACTGAA	67
IGF1R	TTCAGCGCTGCTGATGTG	AAGTTCCCGGCTCATGGT	7
MCL1	AAGCCAATGGGCAGGTCT	TGTCCAGTTTCCGAAGCAT	4
NOL3	AGGCTGAAGCAGAACCAGAG	GCTCTGGCCTTCAGGAATC	36
SPATA2	TCGCTCAGCTCCTCTAGCC	AGGGCCCGTGTAGGTCTT	67
SYCP2	ACCAATTCCACGACCACTGT	GTTTTGTGTTTCTTGTGTAAGTACCAG	61
TGFβ1	ACTACTACGCCAAGGAGGTCAC	TGCTTGAACTTGTCATAGATTTCG	31
TNF	CAGCCTCTTCTCCTTCCTGAT	GCCAGAGGGCTGATTAGAGA	29
TNFRSF11B	GAAGGGCGCTACCTTGAGAT	GCAAACTGTATTTCGCTCTGG	17
TNFRSF1A	CTGCTCCAAATGCCGAAA	CGGTCCACTGTGCAAGAAG	39
TNFRSF10A	GGAGGCACAGTGTCTGCTG	CAGCACCATTTGCTGGAAC	29
TP53	AGGCCTTGGAACTCAAGGAT	CCCTTTTTGGACTTCAGGTG	36
TRAF2	AAGTTCCCCTTAACTTGTGACG	CAAGTCTTGACGTGGTCCTG	7
XIAP	TTTTGGGACATGGATATACTCAGTT	AGCACTTTACTTTATCACCTTCACC	68

* Probe number in Universal ProbeLibrary.

### Western blot analysis

Total proteins were extracted using lysis buffer containing RIPA (1% Nonidet P-40, 0.1% SDS sodium dodecyl sulfate and 0.5% sodium deoxycholate), 1% phenylmethylsulfonyl fluoride (Roche Applied Science) and 2% protease inhibitor cocktail (Roche Applied Science). Protein concentrations were determined by BCA Protein Assay Kit (Pierce Biotechnology). Proteins were separated in polyacrylamide gel, transferred to polyvinylidene difluoride (PVDF) membrane (Millipore), and hybridized with anti-GFPT1 antibodies (Abcam) and anti-TGFβ1 antibodies (Abcam). Hybridization signals were examined using ECL Plus Western Blotting Detection Reagents (Amersham Biosciences).

### Immunocytochemistry

Cells seeded on chamber slide were washed with PBS and fixed with 4% paraformaldehyde. Cells were incubated with anti-GFPT1 antibodies (Abcam) or anti-TGFβ1 antibodies (Santa Cruz Biotechnology) and CF™488A Secondary Antibody Conjugates (Biotium). The nucleus was stained by blue-fluorescent DAPI (Life Technologies). Slides were observed and analyzed with the fluorescence microscope (Nikon).

### Luciferase reporter assay

Sense and anti-sense strands of 2 different regions of 3′-UTR of GFPT1 harboring the binding sites of ebv-miR-BART7 were synthesized. The two single strands of 3′-UTR of GFPT1 containing mutation in the binding sites of ebv-miR-BART7 were also synthesized. The sequences of wild-type and mutant 3′-UTR of GFPT1 were listed in Table 2. After annealing, the sense and anti-sense strands of wild-type or mutant 3′-UTR of GTPT1 were cloned into the SacI and HindIII sites of pMIR-REPORT Luciferase vector (Life Technologies). HONE1 cells were co-transfected with ebv-miR-BART7 mimics or negative control (Qiagen), luciferase vector containing wild-type or mutant 3′-UTR of GTPT1 as well as pMIR-REPORT β-galactosidase control vector (Life Technologies). Dual-Light luminescent reporter gene assay kit (Life Technologies) was employed to determine the firefly luciferase and β-galactosidase activities on a LB 96V microplate luminometer (EG & G Berthold).

**Table 2:** The sequences of wild-type and mutant 3′-UTR of GFPT1 harboring the binding sites of ebv-miR-BART7

Region	Sense strand (5′–3′)	Anti-sense strand (5′–3′)
Wild-type 3′-UTR(15 bp-36 bp)	CGGAATATCTATACAAAATGTACGAAACT GTATGATTAAGCAACACAAGACA	AGCTTGTCTTGTGTTGCTTAATCATACAGT TTCGTACATTTTGTATAGATATTCCGAGCT
Mutant 3′-UTR(15 bp-36 bp)	CGGAATATCTATACAAAATGTACGAAACT GCCGAGCTAAGCAACACAAGACA	AGCTTGTCTTGTGTTGCTTAGCTCGGCAG TTTCGTACATTTTGTATAGATATTCCGAGCT
Wild-type 3′-UTR(1856 bp-1877 bp)	CGGGTTTGTAGCATTTCTTATAGTTTAAA GTATGATTCAGCATTCTAAGTTA	AGCTTAACTTAGAATGCTGAATCATACTTT AAACTATAAGAAATGCTACAAACCCGAGCT
Mutant 3′-UTR(1856 bp-1877 bp)	CGGGTTTGTAGCATTTCTTATAGTTTAAA GCCGAGCTCAGCATTCTAAGTTA	AGCTTAACTTAGAATGCTGAGCTCGGCTTTA AACTATAAGAAATGCTACAAACCCGAGCT

### Colony formation assay

Cells were irradiated at a single dose of 2, 4, 6 or 8 Gy and were seeded in a six-well plate with 600 cells in each well. After incubation for 14 days, cells were fixed with 70% ethanol and stained with 0.5% crystal violet. The numbers of colonies with more than 50 cells were counted.

### Proliferation assay

RTCA DP instrument of xCELLigence Real-Time Cell Analyzer (Roche Applied Science) was used to examine proliferation of NPC cells. Cells were seeded on E-Plate 16 and were irradiated by Gammacell^®^ 3000 Elansystem (Best Theratronics Ltd.). The proliferation rate of NPC cells was monitored continuously and expressed as cell indexes.

### H2AX phosphorylation detection

NPC cells were seeded on chamber slides and irradiated at a single dose of 2, 4, 6 or 8 Gy. Then, cells were washed, fixed and incubated with rabbit polyclonal anti-γH2AX antibodies (Abcam) and CF™488A Secondary Antibody Conjugates (Biotium). The nucleus was stained by DAPI (Life Technologies). F-actin was stained by Alexa Fluor^®^ 635 phalloidin (Life Technologies). Slides were observed with the fluorescence microscope (Nikon) and the number of γH2AX foci in the cells was counted.

### Acridine orange (AO) staining

Formation of acidic vesicular organelles (AVO) during autophagy process is detected by AO staining (Sigma). AVO will appear as fluoresced red, while the cytoplasm and nucleolus are fluoresced bright green and dim red. Stained cells were observed using a fluorescent microscope (Nikon) or analyzed using Cytomics^™^ FC 500 (Beckman Coulter). The percentage of cells undergoing autophagy was calculated.

### Cell apoptosis by annexin V staining

Apoptotic cells were detected by Annexin V staining on the BD FACSCalibur Flow Cytometry System (BD Biosciences). Cells were irradiated, harvested, and resuspended in Annexin-binding buffer (10 mM HEPES, 140 mM NaCl and 2.5 mM CaCl_2_, pH 7.4). Then, cells with stained with APC-labelled Annexin V (BD Biosciences) and 7-AAD (BD Biosciences) and analyzed by flow cytometry.

### Animals

Embryo of wild type zebrafish AB line were maintained in standard zebrafish E3 embryo medium and irradiated following procedure described [34]. Cell irradiation was performed using Gammacell^®^ 3000 Elansystem (Best Theratronics Ltd.) at 4 hpf (hour post fertilization). The embryo was incubated in TGFβ1 (10 ng/ml) containing E3 medium for 2 hours before radiation treatment. Continued embryo survival was assessed by cardiac contractility up to 9 days. Percentage survival was defined as the ratio between irradiated group and non-irradiated group (20 embryos per group). The study was approved by the Institutional Animal Use in Research Committee at the University of Hong Kong (reference number: 2939-13).

### Overexpression of GFPT1 by lentivirus infection

Full-length GFPT1 coding sequence was cloned into lentiviral expression vector pCDH (System Biosciences) to generate the GFPT1-expressing vector. Vector sequences were verified by direct sequencing. Virus packaging was performed by transient transfection into 293T cells using Lipofectamine^®^ 2000 Transfection Reagent (Invitrogen). NPC cell lines HONE1 and HK1 were transduced with medium containing lentivirus.

### Statistical analysis

All the tests were performed using SPSS software version 20.0. All the tests were 2-sided. *P value* < 0.05 was considered as statistical significant.

## Abbreviations

AO: Acridine orange
AVO: Acidic vesicular organelles
EBV: Epstein-Barr virus
Ebv-miR-BART7: EBV-encoded microRNA BART7
NPC: Nasopharyngeal carcinoma
GFPT1: Glutamine-fructose-6-phosphate transaminase 1
TGFβ1: Transforming growth factor beta 1
XIAP: X-linked inhibitor of apoptosis protein
